# Influence of Intraocular Lens Asphericity and Blue Light Filtering on Visual Outcome, Contrast Sensitivity, and Aberrometry after Uneventful Cataract Extraction

**DOI:** 10.18502/jovr.v15i3.7449

**Published:** 2020-07-29

**Authors:** Argyrios Tzamalis, Myron Kynigopoulos, Grigoris Pallas, Ioannis Tsinopoulos, Nikolaos Ziakas

**Affiliations:** ^1^2nd Department of Ophthalmology, Aristotle University of Thessaloniki, Papageorgiou General Hospital, Thessaloniki, Greece; ^2^Department of Ophthalmology, Clinic Pallas, Olten, Switzerland

**Keywords:** Aberrometry, Asphericity, Blue-light Filtering, Contrast Sensitivity, Intraocular Lens

## Abstract

**Purpose:**

To evaluate the effect of asphericity and blue light filter (BLF) of three different intraocular lenses (IOLs) on the visual performance, second- and third-order aberrations (defocus, coma, trefoil), and contrast sensitivity after uneventful cataract surgery.

**Methods:**

One hundred and twenty eyes of 60 patients with clinically significant cataract were randomly assigned to receive one of the three IOL types: Bioline Yellow Accurate (aspheric, with BLF, i-medical, Germany), BioAcryl 60125 (spherical, without BLF, Biotech, France), and H65C/N (aspheric, without BLF, PhysIOL, Belgium). Each IOL was implanted in 40 eyes. Complete ophthalmologic examination, functional acuity contrast testing and wavefront analysis were performed 60 days postoperatively.

**Results:**

The mean postoperative best-corrected visual acuity (BCVA) was 0.95 ± 0.08, not differing statistically among the IOL groups (*P* = 0.83). Mean defocus and coma values did not yield any statistically significant difference through the IOL groups varying from –0.784 to –0.614 μm and 0.129 to 0.198 μm (*P* = 0.79 and 0.34, respectively). Bioline Yellow Accurate IOL presented less trefoil aberrations, 0.108 ± 0.05 μm, compared to the other two IOL types (BioAcryl [0.206 ± 0.19 μm] and Physiol [0.193 ± 0.17 μm], *P* < 0.05). Contrast sensitivity values did not differ among the groups under all lighting conditions. Bioline Yellow IOL showed a statistically higher loss of contrast sensitivity (between mesopic and mesopic with glare conditions) compared to the BioAcryl and PhysIOL in 12 and 3 cpd spatial frequencies, respectively (*P* < 0.05).

**Conclusion:**

Bioline Yellow IOL indicated lower contrast sensitivity under mesopic conditions when glare was applied but resulted in less trefoil aberrations after uneventful cataract surgery. No further differences were noted in postoperative visual performance among three IOL groups.

##  INTRODUCTION

Modern cataract surgery has recently evolved into a refractive procedure aimed at improving visual quality in addition to increasing the visual acuity. Therefore, it is routinely combined with the implantation of intraocular lenses (IOLs) of various materials and designs.

Since the initial introduction of IOLs, there has been great debate over the importance of light filtration.^[1,2,3,4,5]^ Ultraviolet (UV) light-filtering lenses have been the dominant IOLs used in the modern era since growing evidence indicated that UV light could result in photic retinopathy and other retinal pathologies.^[6]^ There has recently though been support for increasing the range of light absorption by IOLs. The rationale was that UV light-filtering IOLs cannot offer protection to the retina from phototoxic damage induced by the high-energy, short-wavelength blue light (400–480 nm), which is considered to contribute to the pathogenesis of age-related macular degeneration (AMD).^[7,8]^ In response to this potential damage, blue light-filtering (BLF) IOLs were introduced in 1996 and have been thereafter widely used, especially in cataract surgery candidates with signs of AMD as a possible measure of preventing associated retinal pathology.^[3,9,10,11,12]^ The yellow tint of BLF IOLs replicates the spectral transmission properties of the aged human crystalline lens in a much closer manner than the UV light-filtering IOLs do.^[13]^

While the possible visual benefits of BLF IOLs are still under debate, controversy has been raised whether a yellow-tinted IOL could modify the visual performance of patients, specifically regarding the postoperative best-corrected visual acuity (BCVA), contrast sensitivity, color vision, and glare.

In addition to BLF, another recently commercialized property of IOLs that has become increasingly popular is asphericity. Spherical aberration has a strong influence on image quality.^[14]^ It is well-established that conventional-spherical IOLs degrade image quality by increasing the spherical higher-order aberrations (HOAs), and several authors have published studies indicating that aspheric IOLs may improve retinal image quality and mesopic contrast sensitivity at low spatial frequencies.^[15,16,17,18,19,20,21]^

However, the combination of BLF and asphericity in IOLs has not been clearly investigated regarding their effect on contrast sensitivity, aberrometry, and quality of vision. The purpose of this prospective randomized study was to evaluate the effect of blue light-filtering and aphericity of IOL on visual quality by comparing the three different IOL types: one aspherical IOL with BLF, one aspherical IOL without BLF, and one spherical IOL without BLF.

##  METHODS

This prospective, randomized clinical study comprised patients who underwent bilateral cataract surgery for visually significant cataract. Sixty patients were randomly assigned to receive one of the three IOL types. Group A received Bioline Yellow Accurate IOL (aspheric with BLF, i-medical, Germany), Group B had H65C/N IOL (aspheric without BLF, PhysIOL, Belgium), and Group C had BioAcryl60125 IOL (spherical, without BLF). Each IOL was implanted in 40 eyes of 20 randomly selected patients. All patients were recruited from the outpatient anterior segment unit of the Clinic Pallas Ophthalmology Department in Olten, Switzerland. The study was performed in adherence with the Declaration of Helsinki for research involving human subjects after approval was obtained from the Institutional Review Boards of Pallas Clinic.

Patients with bilateral cataract with visual disturbance and no history of color vision deficiency were eligible for inclusion in the study. The exclusion criteria were ocular diseases such as corneal opacity or irregularity, astigmatism greater than 2.5 D, dry eye syndrome, inadequate visualization of the fundus, amblyopia, anisometropia, calculated IOL power less than 10.0 diopters (D) or more than 30.0 D, surgical complications, IOL tilt, previous or current use of medications known to cause color vision deficiencies, and incomplete follow-up. Also, patients with a history of uveitis and current intraocular inflammation, uncontrollable glaucoma, proliferative diabetic retinopathy, or retinal detachment were excluded from the study.

One experienced surgeon (GP) performed all surgeries with standard small incision phacoemulsification and IOL implantation in the capsular bag. The time between first eye surgery and second eye surgery was six–eight days in all cases. All eyes were targeted for emmetropia. Table 1 shows the characteristics of the IOLs implanted during the study period. All patients were given combined antibiotic–steroid eye drops for four weeks postoperatively. Patients were examined before surgery and one, seven, and one to three months postoperatively. At all visits, the best corrected-distance visual acuity (BCDVA) and uncorrected distance visual acuity (UDVA) were measured. Contrast sensitivity assessment and aberrometry by means of wavefront analysis were evaluated at the baseline (preoperatively) and last follow-up visit which was performed one to three months postoperatively.

**Table 1:** Main characteristics and special features of the intraocular lenses used in the study


	**Bioline Yellow**	**H65C/N**	**Bioacryl**
**Optic material**	Hydrophilic acrylic copolymer 26 % water	Hydrophilic acrylic copolymer	Hydrophilic acrylic
**Optic design**	Biconvex aspherical	Biconvex aspherical	Spherical
**Optic diameter (mm)**	6.0	6.5	6.0
**Length (mm)**	12.0	12.5	12.5
**Design**	360º square edge	360º reinforced edge design "pco-barrier"	360º square edge
**Haptic angulation (º)**	0	5	5
**Ultraviolet filter**	With BLF	Without BLF	Without BLF
**A-constant**	118.8	118.9	118.0
**Refractive index**	1.465	1.46	1.462
**Estimated anterior chamber depth (mm)**	4.98	4.99	4.96
BLF, blue light filtering			

**Table 2:** Main demographics and clinical characteristics of the study participants


	**Bioline Yellow**	**H65C/N**	**Bioacryl 60125**	**** ***P*** **-value†**	**Total**
**Gender**	21F/19M	22/18M	19F/21M	0.79	62F/58M
**Age (years)**	70.3 ± 8.7	74.6 ± 8.3	72.5 ± 6.4	0.53	72.4 ± 9.5
**Axial length (mm)**	24.1 ± 1.7	23.6 ± 2.2	23.7 ± 1.6	0.59	23.8 ± 2.1
**Preoperative SE**	0.19 ± 1.4	0.45 ± 1.5	0.39 ± 1.2	0.68	0.34 ± 1.5
**Postoperative SE**	–0.32 ± 0.2	–0.34 ± 0.2	–0.31 ± 0.2	0.41	–0.32 ± 0.4
**IOL power**	21.1 ± 2.9	22.5 ± 1.4	22.2 ± 2.6	0.15	21.8 ± 2.5
**BCVA preop, LogMAR (Decimal)**	0.38 (0.42 ± 0.16)	0.29 (0.53 ± 0.12)	0.32 (0.48 ± 0.18)	0.59	0.33 (0.47 ± 0.17)
**BCVA postop, LogMAR (Decimal)**	0.018 (0.96 ± 0.07)	0.031 (0.93 ± 0.09)	0.022 (0.95 ± 0.08)	0.59	0.023 (0.948 ± 0.08)
**CR preop**	7.65 ± 0.3	7.64 ± 0.3	7.66 ± 0.2	0.87	7.65 ± 0.3
**CR postop**	7.66 ± 0.3	7.66 ± 0.3	7.69 ± 0.2	0.93	7.67 ± 0.3
† *P*-value (significance level) was calculated by means of chi-square test, ANOVA-test and Kruskal–Wallis test. SE, spherical equivalent; BCVA, best-corrected visual acuity; CR, corneal radius LogMAR, logarithm of the minimum angle of resolution					

**Table 3:** Comparisons of second- and third-order aberrations among the three IOL groups


**IOL (Group)**	**Defocus (Z200)**	**Coma (Z311, Z310)**	**Trefoil (Z331, Z330)**
**Bioline Yellow (Group1)**	–0.7842 ± 0.9915	0.1288 ± 0.1075	0.1082 ± 0.049
**PhysIOL H65C/N (Group2)**	–0.6557 ± 0.7235	0.1573 ± 0.1186	0.1929 ± 0.1736
**BioAcryl 60125 (Group3)**	–0.6136 ± 0.6239	0.1984 ± 0.1599	0.2058 ± 0.1852
**Mean**	–0.69 ± 0.72	0.16 ± 0.13	0.16 ± 0.15
**Comparison between groups (** ***P*** **-value)**
**Group1–Group2**	0.52	0.11	**0.03**
**Group1–Group3**	0.59	0.43	**0.042**
**Group2–Group3**	0.8	0.36	0.82

**Table 4:** Mean loss of contrast sensitivity, expressed in logarithmic units, from photopic to mesopic lighting conditions (LC) and under glare conditions in mesopic LC.


	**Loss Photopic to Mesopic LC**					**Loss Mesopic to Mesopic with glare LC**				
**Spatial Frequency (cpd)**	**1.5**	**3**	**6**	**12**	**18**	**1.5**	**3**	**6**	**12**	**18**
**IOL1**	0.051	0.105	0.227	0.406	0.569	0.096	0.23	0.233	0.452	0.256
**IOL2**	0.021	0.129	0.185	0.400	0.358	0.165	0.121	0.154	0.217	0.331
**IOL3**	0.030	0.140	0.221	0.521	0.433	0.167	0.139	0.229	0.099	0.206
**** ***P*** **(ANOVA)†**	0.69	0.62	0.69	0.69	0.27	0.17	0.04	0.75	0.02	0.64
**** ***P*** **(** ***t*** -test)1–2*	0.43	0.59	0.17	0.77	0.25	0.13	0.01	0.71	0.27	0.84
**** ***P*** **(** ***t*** -test)1–3*	0.53	0.31	0.60	0.34	0.41	0.20	0.05	0.84	0.04	0.81
**** ***P*** **(** ***t*** -test)2–3*	0.92	0.64	0.60	0.62	0.75	0.98	0.77	0.85	0.30	0.67
IOL1, Bioline Yellow Accurate; IOL2, Physiol H65C/N; IOL3, BioAcryl60125; cpd, cycles per degree † *P*-value comparing all three IOL groups with one-way analysis of variance; *P-value comparing IOL groups in couples with Student's *t*-test										

Visual acuity was measured using Snellen chart under scotopic conditions (target luminance 1.5 candelas [cd]/m2). Contrast sensitivity was assessed using Functional Acuity Contrast Testing (FACT-Optec6500, Stereo Optical Inc., USA) with spectacle correction under photopic conditions (target luminance value 85 candelas [cd]/m2) and mesopic conditions (target luminance value 3 cd/m${}^{2}$) with and without glare. Lighting conditions were controlled with a luxometer (Gossen-Starlite). The log base 10 contrast sensitivity values were used to construct a graph for each spatial frequency tested and then presented using the original test scale.

A Zywave Hartmann-Shack aberrometer (Bausch & Lomb, Germany) was used for all aberrometry measurements. Zywave was used to assess and compensate for the refractive errors, and, eye fogging system was acquired before each wavefront measurement to avoid patient accommodation. Before use, the aberrometer was calibrated by an experienced Bausch & Lomb technician to ensure the accuracy. Five measurements were performed by a single experienced technician to avoid interobserver variability in the results; of these, two measurements with higher deviations from the mean were excluded and the three best measurements were averaged and used for statistical analyses. Patients were instructed to blink between measurements, and acquisition was obtained after a blink to ensure higher quality results by limiting tear film disruption. All results were exported as raw data so that individual Zernike terms could be analyzed independently. The details of the Zernike coefficients up to the third order were recorded and used for the statistical analysis. Zywave measurements were obtained without any pharmacologic mydriasis and dark adaptation. Nonetheless, all measurements were made in certain mesopic lighting conditions and it was confirmed that pupillary diameter was at least 6 mm in every case. Total, corneal, and internal components for each of the high-order aberrations were obtained and used for the analysis.

Statistical analysis was performed using the SPSS (version 17.0 for Windows, SPSS, Inc. Chicago, IL) and MedCalc statistical software (version 9.3.0.0, MariaKerke, Belgium). Normality was checked using the Kolmogorov–Smirnov test. Since data were not normally distributed in all cases, both parametric and nonparametric methods were used. For normally distributed data, Pearson correlation was used to evaluate the association between two continuous variables and the one-way analysis of variance (ANOVA) was applied to evaluate the influence of a qualitative factor on another continuous variable. The association of not normally distributed data was assessed using Rank correlation calculating Spearman's coefficient rho. When parametric analysis was possible, the Student's *t*-test was used to compare the outcomes between two IOL groups. Categorical variables were compared using the Fisher's exact test. Nonparametric Kruskal–Wallis and Mann–Whitney tests were also used to examine the associations between categorical variables and continuous or ordered outcomes. A *P*-value of < 0.05 was defined for all statistical tests as statistically significant.

##  RESULTS 

A total number of 120 eyes of 60 patients (mean age, 72.4 ± 9.5 years) who underwent uneventful bilateral cataract surgery were found eligible and were finally enrolled in the statistical analysis. All eyes were divided into one of the three groups, based on the type of IOL they received. The main demographic and clinical characteristics of each group are demonstrated in Table 2.

There were no statistically significant differences among the study groups in preoperative clinical and refractive values. Preoperative total and internal components of aberrometry showed great deviations between cases, as patients with various degrees and types of cataracts were included. However, the preoperative corneal component of coma, defocus, and trefoil did not have any statistically significant difference among the three groups (*P* > 0.05). The mean Snellen postoperative BCVA was 0.95 ± 0.08 (0.023 LogMAR) with a mean postoperative spherical equivalent of –0.32 ± 0.13D; not differing statistically between the IOL groups (Table 2; *P* > 0.05). The mean LogMAR uncorrected VA (UCVA) increased from 0.58 ± 0.25 (Bioline Yellow), 0.54 ± 0.23 (H65C/N), and 0.55 ± 0.26 at screening to 0.23 ± 0.12, 0.22 ± 0.11, and 0.24 ± 0.11, respectively, at postoperative follow-up. There was no statistically significant difference in the postoperative UCVA among the IOL groups. Postoperative values were recorded at a mean time of 63.2 ±11.7 days after uneventful cataract surgery varying between 38 and 87 days, with no significant difference among the groups in the duration of follow-up (ANOVA, *P* = 0.21). Table 2 demonstrates the preoperative and postoperative refraction data in more details.

Mean defocus and coma values did not yield any statistically significant difference among IOL groups varying from –0.784 to –0.614 and 0.129 to 0.198, respectively (Table 3). Bioline Yellow Accurate presented less trefoil aberrations, 0.108 ± 0.05 μm compared to the other two IOL types (*P* < 0.05). Table 3 illustrates the second- and third-order aberrations as well as their intergroup comparisons.

**Figure 1:**
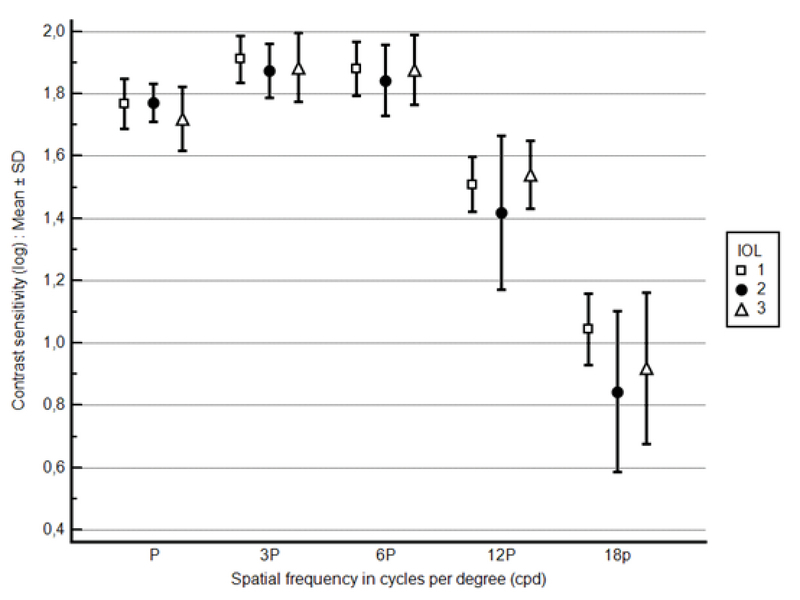
Postoperative contrast sensitivity (log) under photopic conditions (85 cd/m2) in various spatial frequencies for the IOLs included in the study [IOL1 = Bioline Yellow Accurate (i-medical, Germany), IOL2 = BioAcryl 60125 (Biotech, France), IOL3 = H65C/N (PhysIOL, Belgium)].

**Figure 2:**
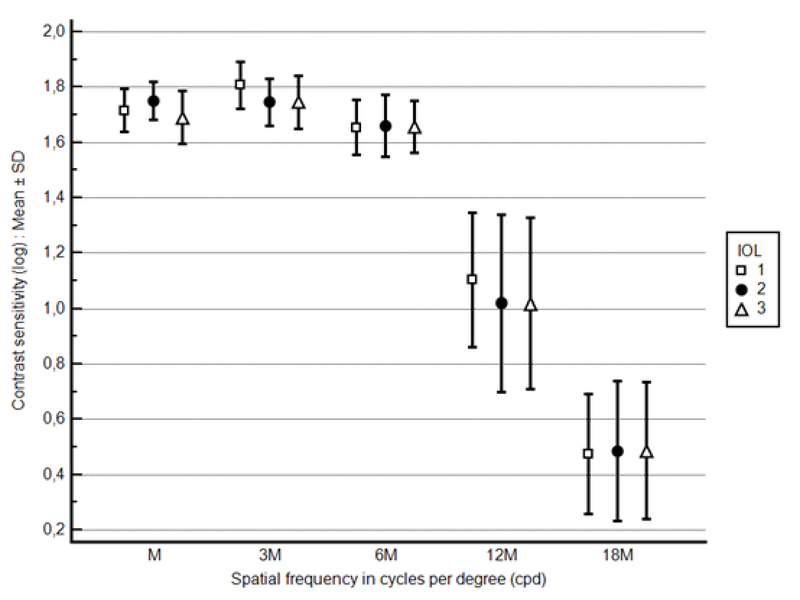
Postoperative contrast sensitivity (log) under mesopic conditions (3 cd/m2) in various spatial frequencies for the IOLs included in the study [IOL1 = Bioline Yellow Accurate (i-medical, Germany), IOL2 = BioAcryl 60125 (Biotech, France), IOL3 = H65C/N (PhysIOL, Belgium)].

**Figure 3:**
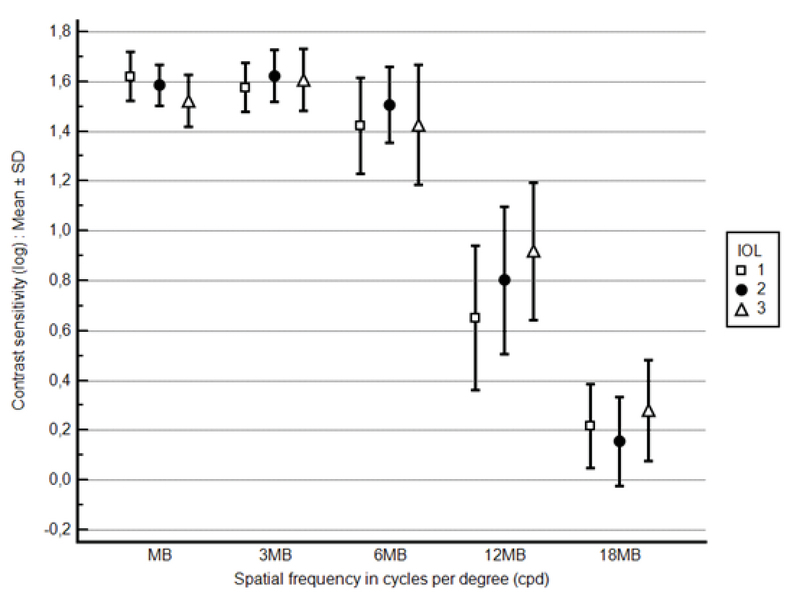
Postoperative contrast sensitivity (log) under mesopic with glare conditions (3 cd/m2 with glare) in various spatial frequencies for the IOLs included in the study [IOL1 = Bioline Yellow Accurate (i-medical, Germany), IOL2 = BioAcryl 60125 (Biotech, France), IOL3 = H65C/N (PhysIOL, Belgium)].

There was no statistically significant difference among the three IOL groups in contrast sensitivity at any spatial frequency under all three lighting conditions. Figure 1 (photopic 85 cd/m2), Figure 2 (mesopic 3 cd/m2), and Figure 3 (mesopic with glare) depict postoperative contrast sensitivity for all IOL groups. In a separate analysis, Bioline Yellow was found to have a statistically lower contrast sensitivity under glare conditions compared to the BioAcryl and PhysIOL in 12 and 3 cpd spatial frequencies, respectively (*P* < 0.05). Table 4 compares the postoperative contrast sensitivity among the IOL groups.

##  DISCUSSION

Modern cataract surgery with the implementation of specially designed IOLs has developed tremendously over the past decades, attempting to meet patients' expectations for optimal visual outcomes.^[11,19,21]^ Contemporary diagnostic tools have extended our knowledge on the impact of HOAs and contrast sensitivity on the quality of vision. Therefore, in order to achieve the best outcome after phacoemulsification, the IOL implantation should result in minimal aberrations and high-contrast sensitivity.

IOLs with aspheric optics, designed to optimize postoperative spherical aberration and implants with BLF as a possible measure of preventing associated retinal pathology have gained great popularity. However, there is still great controversy on their potential benefit and the effect of these features on the postoperative visual performance, specifically regarding the ultimate BCVA, contrast sensitivity, color vision, and postoperative aberrations.^[1,2,3,4,5][9][10][11][12][15,16,17,18,19,20,21]^

The present prospective randomized study attempted to investigate the effect of BLF and aspherical IOL design on the final visual outcome. Therefore, we compared the visual performance after the implantation of three different IOLs; one aspheric, with BLF; one aspheric, without BLF; and one spherical, without BLF. Our results showed that BCVA did not differ statistically significantly among the IOL groups. These results regarding the effect of BLF in postoperative BCVA are in concordance with a recent Cochrane Database Systematic Review which demonstrated, with moderate certainty, that the presence of BLF in IOLs had no clinically meaningful effect on short-term BCVA.^[22]^

Although no significant difference was found in our study among the different IOL groups in the postoperative BCVA, the group of patients implanted with an aspheric IOL with BLF indicated fewer trefoil aberrations when compared to the other IOL groups included in the study. Notably, the preoperative corneal component of HOAs did not differ significantly among the three IOL groups. Therefore, the lower trefoil measurements shown in this group could be attributed to the internal components, mainly the IOL itself. A postoperative IOL tilt could also be a predisposing factor for increased aberrations.

Blue-light filtering is an add-on feature of IOLs, considered to offer an extra retinal protection against AMD, although this has not been fully proven so far.^[23]^ IOLs with BLF are supposed to reduce longitudinal chromatic aberrations. Theoretically, such a reduction should not affect spherical aberrations. However, in our study, the yellow-tinted IOL achieved better results in postoperative trefoil when compared not only to the spherical IOL but also to the aspheric one without BLF. It should be noticed that the two aspheric IOLs used in this study had minimal differences in terms of optical design and material being produced by different manufacturers. This fact could also have some impact on the results reported. To the best of our knowledge, there are no published studies evaluating the effect of BLF on spherical HOAs by comparing the same IOL types.

As far as postoperative contrast sensitivity is concerned, no significant difference was found among IOLs in any spatial frequency under photopic, mesopic, and mesopic with glare-lighting conditions. However, the yellow-tinted aspheric IOL was found to have a statistically higher loss of contrast sensitivity under glare conditions compared to the non-tinted IOLs at some spatial frequencies.

In recent years, aspheric IOLs have gained increasing popularity among surgeons due to their theoretical advantage of being able to compensate for the spherical aberration of the human cornea, with the aim of restoring the optical performance of the eye.^[14]^ Most studies performed on this task have confirmed this theory reporting that aspheric IOLs implanted have significantly reduced the overall spherical aberrations, hence improving optical performance in certain cases.^[15,17,18,19,20,21,22,23,24]^

Comparing the aspheric Tecnis ZA9003 IOL with the spherical AcrySof SA60AT IOL (Alcon, Inc.), Kim et al^[25]^ reported a significant improvement in contrast sensitivity under mesopic and photopic conditions with the aspheric IOL; the authors reported that the mean spherical aberration was significantly higher in eyes implanted with the spherical IOL, although total higher order aberrations did not differ significantly between the results of two further prospective randomized studies performed by Rocha et al and Caporrosi et al, who concluded that eyes implanted with aspheric IOLs had less aberrations and performed better under mesopic condition compared to spherical IOLs.^[18,26]^

On the contrary, several researchers have reported no statistically significant differences in visual acuity and contrast sensitivity between spherical IOLs and aspheric IOLs.^[27,28,29]^ We compared in our study the outcome of the three different IOLs, two aspheric and one spherical and found no statistically significant difference in the second- and third-order aberrations other than trefoil aberrations that was lower in the eyes implanted with aspheric IOL with a BLF. Surprisingly, no difference was noted between the two non-tinted IOL groups, despite one of them having an aspherical design. One may hypothesize that BLF added on yellow-tinted IOLs could reduce some spherical aberrations along with the longitudinal chromatic ones; however, this theory needs to be examined by further prospective randomized studies with larger population sizes to compare aberrations between IOLs of identical design and material.

In the past decade, several manufacturers and distributors have promoted commercially available IOLs with BLF properties. Theoretically, BLF IOLs may induce a reduction in mesopic and scotopic visual performance attributed to the Purkinje shift, where differing peaks of spectral sensitivity for scotopic and photopic vision are identified.^[1,3]^ Violet and blue lights are much more important for vision in dim-light environments than in bright-light environments, providing 45% of rod-mediated aphakic scotopic sensitivity but only 7% of photopic sensitivity for an iso-illuminance light source.^[1,2]^ However, the results reported in the literature are controversial regarding postoperative contrast sensitivity after the BLF-IOL implantation and do not indicate a significant decrease in the mesopic and scotopic visual function.

Kara-Junior et al^[30]^ investigated the long-term possible side effects after implantation of an IOL with a BLF. The authors found no significant differences in color perception, scotopic contrast sensitivity, or photopic contrast sensitivity between the BLF IOL and the IOL with a UV-light filter only. In another study, Greenstein et al^[31]^ investigated contrast sensitivity in nine patients implanted with a BLF IOL (AcrySof SN60AT) in one eye and a UV-only filtering IOL (AcrySof SA60AT) in the fellow eye. In addition, they compared the results with those obtained in nine young phakic patients and found no significant difference in hue discrimination or dark-adapted sensitivity between the two IOLs.^[31]^ These results were comparable to the outcome of a study by Muftuoglu et al^[32]^ who compared photopic and scotopic CS in eyes with an AcrySof SN60AT IOL (with BLF) and eyes with a conventional AcrySof SA60AT IOL (UV-only filtering) and reported no statistically significant differences between the two IOL types. Furthermore, Hayashi et al^[9]^ measured contrast visual acuity in 74 patients implanted bilaterally with either tinted IOL (HOYA YA60BB) or non-tinted IOLs (VA60BB) and reported no significant difference between the IOL groups.

In concordance with these aforementioned studies, our results showed no statistically significant difference in contrast sensitivity between IOLs with and without BLF. In an additional analysis, we evaluated the loss of contrast sensitivity after glare was applied in mesopic conditions and found that the tinted IOL had a statistically greater loss of contrast sensitivity under glare compared to the non-tinted IOLs, but only in some spatial frequencies. Although this may be an accidental finding, it is noteworthy as most previous studies did not include contrast sensitivity measurement in mesopic conditions under glare, a situation that is rather common in real life, such as night driving, and can substantially affect the patient's quality of life after cataract surgery.

A weakness worth mentioning of all studies reporting mesopic CS results after the implantation of IOLs with BLF is the fact that all have utilized measures that are a function of only central vision, where macular pigment is also acting as a BLF. Moreover, one should consider that mesopic vision is mediated, at least in part, by cones, and therefore it is less likely to be adversely influenced by the transmittance properties of such blue-blocking IOLs. Other limitations of our study include the relatively small sample size and the lack of a group with implantation of a spherical IOL with BLF; this type of IOL was not commercially available at the time the study was conducted.

In summary, the present study compared three IOLs varying in terms of asphericity and BLF and showed only minimal differences in postoperative contrast sensitivity and aberrometry. All IOLs achieved comparable results in postoperative visual performance; an aspheric IOL with BLF, however, resulted in less trefoil aberrations and a greater loss of contrast sensitivity in mesopic conditions when glare was applied. Further randomized patient-centered studies are needed to evaluate the long-term results of aspheric IOL design and BLF and to investigate whether these features are desirable for the patients' quality of life.

##  Financial Support and Sponsorship

Nil.

##  Conflicts of Interest

There are no conflicts of interest.
